# An overview of systematic reviews of acupuncture for diabetic gastroparesis

**DOI:** 10.3389/fmed.2023.1196357

**Published:** 2023-07-31

**Authors:** Ting Li, Mingchen Yu, Lulu Han, Bo Feng, Fenglei Sun

**Affiliations:** ^1^The First Clinical School of Shandong University of Chinese Medicine, Jinan, China; ^2^Rizhao Hospital of Traditional Chinese Medicine, Rizhao, Shandong, China; ^3^The Fifth People’s Hospital of Jinan City, Jinan, Shandong, China; ^4^The First Affiliated Hospital of Shandong First Medical University, Jinan, China; ^5^Affiliated Hospital of Shandong University of Chinese Medicine, Jinan, China

**Keywords:** acupuncture, diabetic gastroparesis, systematic review, overview, meta-analysis

## Abstract

**Background:**

To date, several systematic reviews and/or meta-analyses (SRs/MAs) on the topic of acupuncture as a treatment for diabetic gastroparesis (DGP) have been published. However, whether acupuncture is an effective and safe treatment for DGP remains controversial. In this study, we aimed to determine whether the methodology and results of previously published SRs/MAs of acupuncture as a treatment for DGP were of sufficient quality to be considered reliable.

**Methods:**

We extensively searched seven databases, including PubMed, EMBASE, Cochrane Library, Web of Science, China National Knowledge, Wan Fang, and Chongqing VIP, for SRs/MAs published before or on September 16, 2022. The SRs/MAs that met the inclusion criteria were evaluated for the quality of the methodology and results using the Assessing the Methodological Quality of Systematic Reviews Two (AMSTAR-2) and Grading of Recommendations, Assessment, Development, and Evaluation (GRADE) tools. A re-meta-analysis of primary outcome indicators was also performed.

**Results:**

Ten SRs/MAs that met the inclusion criteria were obtained. Using the AMSTAR-2, which is a methodological quality assessment tool, two MAs were rated as low quality, and eight SRs/MAs were rated as extremely low quality. Assessment with the GRADE tool revealed that, among 20 results, 4 were of moderate quality, 10 were of low quality, and 6 were of very low quality. Re-meta-analysis of primary outcome indicators revealed that, in terms of total efficiency, all types of acupuncture interventions, such as acupuncture, electroacupuncture, and acupoint injection, performed better than the controls, such as gastroprokinetic agents and sham acupuncture. Moreover, in the treatment of DGP, acupuncture exhibited fewer side effects compared to the controls.

**Conclusion:**

Acupuncture appears to improve the symptoms of patients with DGP, and the side effects of acupuncture as a treatment for DGP are inferior to those of the controls. However, owing to the low quality of the methodology and results of the SRs/MAs, these findings cannot be considered reliable and need to be validated by additional studies with rigorous standards of experimental design and protocols and larger sample sizes.

## 1. Introduction

Diabetic gastroparesis (DGP) is a common complication of diabetes, with delayed gastric emptying as its main feature (1). Clinical manifestations include nausea, vomiting, stomach pain, and abdominal distension, which have a notable impact on the quality of life of patients (2, 3). The prevalence of DGP is approximately 40% in patients with type 1 diabetes and approximately 30% in patients with type 2 diabetes (4). The pathogenesis of DGP is not fully understood; however, mechanistic studies suggest that it is mainly associated with autonomic disorders, enteric neuropathy, apoptosis of interstitial cells of Cajal (ICC), abnormal secretion of gastrointestinal hormones, and gastric smooth muscle lesions caused by elevated blood glucose levels (5).

The American Gastroenterological Association 2013 Guidelines recommend prokinetic drugs for the first-line treatment of DGP (6). Studies have shown that metoclopramide, domperidone, tiapride, and erythromycin improve the symptoms of DGP to a certain extent (7–10). However, these drugs inevitably result in a variety of side effects, including nervous system damage, twitching, muscle tremors, irritability, and to a certain extent, respiratory, urinary, and even cardiac arrhythmias (11).

Traditional Chinese medicine, in particular acupuncture, has gained increasing attention in many countries over the past few years. Acupuncture has been shown to be effective in treating shingles, depression, and chronic pain with few side effects (12–14). Acupuncture has also been shown to be an effective treatment for DGP and is increasingly being used as a complementary or alternative treatment.

The efficacy and safety of acupuncture for the treatment of DGP have been the subject of many systematic reviews and meta-analyses (SRs/MAs); however, evidence quality of the SRs/MAs and the methodology of the SRs/MAs have several flaws. Overviewing SRs/MAs is a burgeoning research approach that combines results reported in several SRs/MAs and resolves inconsistencies in the results to provide accurate clinical recommendations (15). Therefore, in this study, we attempted to comprehensively assess SRs/MAs of acupuncture as a treatment for DGP using the Assessing the Methodological Quality of Systematic Reviews Two (AMSTAR-2) and Grading of Recommendations, Assessment, Development, and Evaluation (GRADE) tools. Our findings may serve as scientific evidence for the effectiveness and safety of acupuncture as a treatment for DGP.

## 2. Methods

### 2.1. Study registration

The protocol for this study was registered in PROSPERO (registration number: CRD42022376495). Since this study is a review, ethical approval was not required.

### 2.2. Search strategy

Two researchers (F.D. and L.L.H.) searched seven databases, namely PubMed, EMBASE, Cochrane Library, Web of Science, China National Knowledge, Wan Fang, and Chongqing VIP, for SRs/MAs published before or on September 16, 2022. Language restrictions were not applied. For the search, we used the following keywords: (“acupuncture” OR “electroacupuncture” OR “warm acupuncture” OR “milli needles” OR “acupoint injection” OR “acupoint catgut embedding”) AND (“diabetic gastroparesis” OR “DGP”) AND (“systematic review” OR “meta-analysis” OR “review”). To avoid omitting relevant SRs/MAs, we additionally explored the references cited in the articles that the search yielded.

### 2.3. Inclusion and exclusion criteria

#### 2.3.1. Criteria for inclusion

The criteria for inclusion of articles were as follows: (1) participants: participants older than 18 years who were diagnosed with DGP, irrespective of gender or race; (2) interventions: acupuncture, traditional acupuncture, electroacupuncture, auricular acupuncture, acupoint injections (AIs), acupuncture alone or in combination with conventional Western medicine/Chinese herbal medicine (CM) as treatment group interventions, irrespective of the frequency and duration of treatment; (3) controls: gastroprokinetic agents (GAs) or sham acupuncture; (4) outcomes: total efficiency as the primary outcome indicator and improvement in gastrointestinal symptoms, motilin secretion, gastrin secretion, gastric-emptying rate, and cure rate as the secondary outcome indicators; and (5) and type of study: SR/MA of clinical randomized controlled trials (RCTs).

#### 2.3.2. Criteria for exclusion

The criteria for exclusion of articles were as follows: (1) Protocol of SR/MA, net meta-analysis, meeting abstracts, guidelines, animal experiments, or non-RCT SR/MA; (2) SR/MA comparing different acupuncture categories; and (3) SR/MA that was published twice or from which data could not be extracted.

### 2.4. Selection of literature and extraction of data

Two researchers (M.C.Y. and L.L.H.) independently screened the literature. Screening-related issues were resolved by consulting a third researcher (T.L.). The articles yielded by the initial search were imported to Note Express software (x86). Duplicate articles were excluded. Then, an initial screening was performed based on the inclusion and exclusion criteria by reading the title and abstract of the articles. Articles potentially eligible for inclusion were read in their entirety to determine if they met the inclusion criteria. Thereafter, two researchers (M.C.Y. and L.L.H.) individually retrieved relevant data from the SRs/MAs that met the inclusion criteria and filled out standardized data extraction forms. The following data were extracted: first author, publication year, type of article, number of studies included in the SR/MA, interventions, controls, outcome indicators, number of individuals in the treatment and control groups for the outcome indicators, quality assessment tools, results of the quality assessment of the original studies, and the main findings.

### 2.5. Quality assessment

Two researchers (M.C.Y. and L.L.H.) assessed the quality of the included SRs/MAs using the AMSTAR-2 tool (16). Assessment-related doubts were resolved by consulting a third researcher (T.L.). AMSTAR-2, the latest version of AMSTAR, is a tool for the systematic evaluation of the methodological quality of SRs/MAs and exhibits good inter-evaluator consistency and practicality (17). AMSTAR-2 has a total of 16 entries, 7 of which, i.e., 2, 4, 7, 9, 11, 13, and 15, are critical, and the remaining 9 are non-critical. Key items include SR/MA registration, the search strategy, a list of excluded literature and the exclusion criteria, an assessment of the risk of bias of the original studies using appropriate evaluation tools, the application of appropriate statistical methods to combine study results, the need to consider the risk of bias of original studies when interpreting or discussing study results, and a reasonable analysis of publication bias and discussion of its possible impact on the results in the case of quantitative analysis (17). AMSTAR-2 cannot provide an overall score to SRs/MAs but rather evaluate the methodological quality of SRs/MAs based on the 16 entries and rate them as high, medium, low, or very low quality. In case of no defect in a critical entry or a defect in a non-critical entry, the SR/MA is rated as “high” quality. In case of more than one non-critical defect, the SR/MA is rated as “moderate” quality. In case of a critical entry defect, with or without a non-critical entry defect, the SR/MA is rated as “low” quality. In case of more than one critical entry defect, with or without non-critical entry defects, the SR/MA is rated as “very low” quality (16).

Two researchers (M.C.Y. and L.L.H.) assessed the quality of the results pertaining to the individual outcome indicators reported in the MAs using the GRADE tool. Assessment-related queries were resolved by consulting a third researcher (T.L.). With the GRADE tool, the MAs were assessed for study limitations, inconsistencies, imprecision, non-directivity, and publication bias. Based on the results, the studies included in the MAs/SRs were rated as high, medium, low, or very low quality (18).

### 2.6. Data analysis

To quantify the effectiveness of acupuncture as a treatment for DGP, we re-analyzed the outcome indicators with a high frequency of occurrence (total efficiency, motilin secretion, gastrin secretion, gastric-emptying rate, and adverse reactions). Given the potential for duplication of RCTs in the included MAs, all the RCTs analyzed in the MAs were listed by two researchers independently (M.C.Y. and L.L.H.), and duplicate RCTs were excluded from the list. In addition, in the case of two articles with the same study population published by the same author, the one with the larger sample size was selected. Odds ratio (OR) was adopted for dichotomous data. Standardized mean difference (SMD) was calculated for continuous variables. For efficacy analysis statistic, 95% confidence interval (CI) was applied. *P* < 0.05 was considered to indicate statistical significance. The data were processed using Stata SE 16 software. Considering the potential clinical heterogeneity in the included studies, a random effects model was used for all analyses.

## 3. Results

### 3.1. Literature search

**Figure 1:**
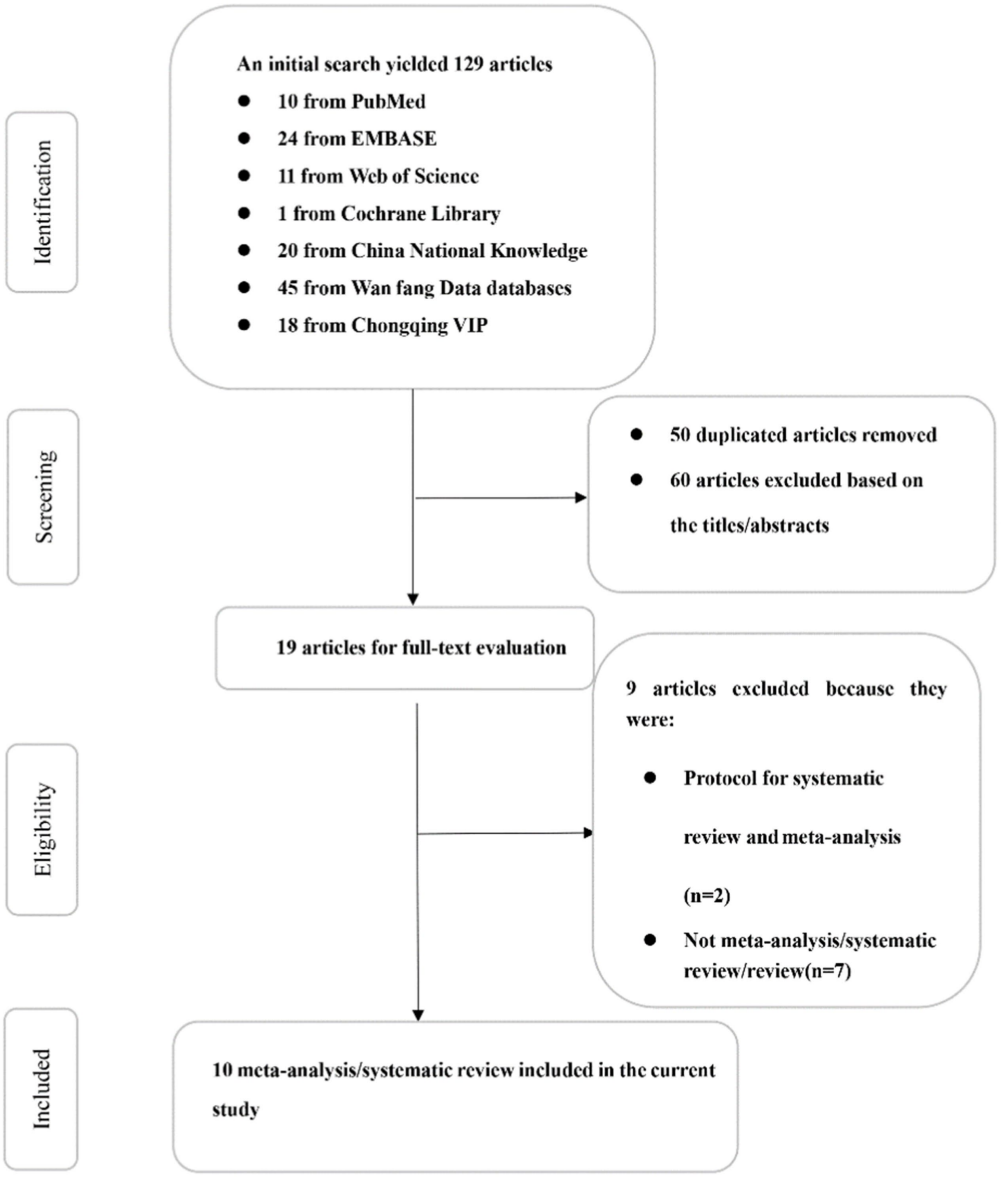
Flow chart of study selection.

Figure 1 shows the flowchart of the literature screening process and its rationale. The database search yielded 129 articles. Next, 50 articles were removed owing to research duplication. Furthermore, 60 additional articles were excluded because the title or abstract was not relevant to the study topic. Subsequently, a total of 19 articles were reviewed in their entirety. Thereafter, two articles were excluded as they reported the protocol of SRs/MAs, and seven were excluded as they were not SRs/MAs. Finally, a total of 10 articles were included in this study.

### 3.2. Features of the included literature

The summary of the data retrieved from the 10 SRs/MAs is shown in Table 1. The articles included in this study, i.e., nine MAs (19–27) and one SR (28), were published from 2010 to 2021 by Chinese authors. The number of RCTs included in the SRs/MAs varied from 9 to 22, with sample sizes ranging from 617 to 1,581 participants. The main interventions analyzed in the SRs/MAs were acupuncture, electroacupuncture, warm acupuncture, AI, acupuncture combined with CM, electroacupuncture combined with CM, and AI combined with CM. Controls were mainly based on sham acupuncture and treatment with Western medicine. There were ten outcome indicators, all of which were related to gastric function and symptom improvement, with total efficiency as the main outcome indicator. Five SRs/MAs had been assessed for quality using the Cochrane risk of bias criteria. The other five SRs/MAs had been evaluated according to the Jadad scale. All of the included studies were of low quality. All ten SRs/MAs reported that acupuncture was effective as a treatment for DGP, but that further validation of the results through high-quality trials was essential. Two SRs/MAs reported that acupuncture reduced motilin and gastrin secretion. Two SRs/MAs reported that acupuncture promoted gastric emptying. However, one article reported that acupuncture had no remarkable impact on gastric emptying.

**Table 1:** Characteristics of the meta-analyses and systematic reviews included in this study.

First author, year (article type)	Trials (sample size)	Treatment	Control	Outcome	Quality of the included studies	Findings
Tang ZM, 2010 (SR)	10 (627)	AC, EA, MN	GA, SA	①②③	Cochrane criteria	Compared to GA/SA, acupuncture was more effective in treating DGP without any adverse reactions. Studies in the literature that met the inclusion criteria were few, and their quality was low. Because the objectivity of the meta-analysis conclusions must be based on high-quality randomized controlled trials, further validation through well-designed, large, and multicenter clinical randomized controlled trials is required.
					a: 8/2 trials with low/unclear ROB	
					b: 10 trials with unclear ROB	
					c: 1/9 trials with low/high ROB	
					d: 10 trials with high ROB	
					e: 1/9 trials with low/unclear ROB	
					f: 10 trials with high ROB	
					g: 10 trials with unclear ROB	
Yang MX, 2013 (MA)	14 (948)	AC, EA, WA	GA, SA	①②④⑦	Cochrane criteria	Compared to GA/SA, acupuncture was more effective in treating DGP but had no notable impact on gastric emptying. However, owing to the low quality of the included trials, no definitive conclusions about the effects of acupuncture on DGP could be drawn. Therefore, well-designed, placebo-controlled, and long-term follow-up trials with larger sample sizes are needed to evaluate the effectiveness of acupuncture as a treatment for DGP.
					a: 8/6 trials with low/unclear ROB	
					b: 14 trials with unclear ROB	
					c: 1/13 trials with low/high ROB	
					d:14 trials with high ROB	
					e: 5/8/1 trials with low/unclear/high ROB	
					f: 7/7 trials with low/unclear trials for ROB	
					g: 1/13 trials with low/unclear ROB	
Wang ZH, 2014 (MA)	9 (678)	AC, WA	GA	①	All the included studies were of low quality (Jadad)	Compared to the GA, acupuncture had a positive effect on the symptoms and signs of DGP. However, because of the low quality of the experimental methodology, the results could not be validated. Therefore, further validation through high-quality trials is required.
He H, 2015 (MA)	10 (755)	AC	GA	①③⑧	Cochrane criteria	Compared to GA, acupuncture exhibited significantly better overall efficacy in the treatment of DGP. However, no significant difference in the improvement of gastrointestinal symptoms was observed between the experimental and control groups. Owing to the small number of studies included in the study and the low quality of the articles, accurate conclusions could not be drawn. Therefore, well-designed, large, and multicenter randomized controlled experiments are required to validate the results.
					a: 1/9 trials with low/unclear ROB	
					b: 10 trials with unclear ROB	
					c: 10 trials with unclear ROB	
					d: 10 trials with low ROB	
					e: 10 trials with low ROB	
					f: 10 trials with unclear ROB	
					g: 10 trials with low ROB	
Zhang H, 2016 (MA)	16 (1189)	AC+CM	GA	①③④	Cochrane criteria	Compared to GA, acupuncture in combination with traditional CM was superior in improving gastric emptying and gastrointestinal symptoms in patients with DGP. However, no remarkable difference in adverse reactions was observed between the experimental and control groups. To obtain more reliable evidence-based medicine, rigorously designed, multicenter, and prospective randomized clinical trials with large sample sizes are necessary in the future.
					a: 5/11 trials with low/unclear ROB	
					b: 16 trials with unclear ROB	
					c: 16 trials with unclear ROB	
					d: 16 trials with unclear ROB	
					e: 16 trials with low ROB	
					f: 16 trials with unclear ROB	
					g: 16 trials with unclear ROB	
Chen YJ, 2018 (MA)	10 (774)	AC, AC+ CM	GA	①	All the included studies were of low quality (Jadad)	Compared to GA, acupuncture exhibited more efficacy in the treatment of type 2 diabetes gastroparesis. However, the literature included in this review was of low quality, and many clinical studies were excluded because they did not employ randomized controlled methods or did not meet statistical criteria. Therefore, future research should be conducted according to the requirements of evidence-based medicine and statistics to avoid low-level study duplication.
Zhang L, 2019 (MA)	9 (698)	AI, AI + CM, AI + GA	GA	①⑤⑥	All the included studies were of low quality (Jadad)	Compared to GA, acupuncture point injection exhibited better overall efficacy in the treatment of DGP and notably reduced the levels of gastrin and motilin in the plasma. However, high-quality clinical studies with large sample sizes and improved methodological quality are required to validate the results.
Liu BY, 2021 (MA)	16 (1403)	AC, AC + GA, EA, EA + GA, WA, WA + GA	GA	①②③⑤⑥⑨	Cochrane criteria	Compared to GA, acupuncture exhibited better overall efficacy in the treatment of DGP, improved the gastrointestinal symptoms of patients, and notably reduced the effect of gastrin and motilin. However, this study lacked long-term follow-up of recurrence rates. Moreover, determining long-term efficacy was difficult.
					a: 6/10 trials with low/unclear ROB	
					b: 16 trials with unclear ROB	
					c: 16 trials with high ROB	
					d: 16 trials with unclear ROB	
					e: 16 trials with low ROB	
					f: 16 trials with unclear ROB	
					g: 16 trials with low ROB	
Chen YF, 2021 (MA)	10 (617)	EA	GA	①②③④⑤⑥	All the included studies were of low quality (Jadad)	Compared to GA, EA exhibited better clinical comprehensive efficacy in the treatment of DGP. However, owing to the low quality of the included literature, multicenter, rigorously designed, randomized, and double-blind controlled clinical trials with large sample sizes should be conducted to provide more objective evidence-based results for the efficacy of EA as a treatment for DGP.
Wang SJ, 2021 (MA)	22 (1581)	AC + CM	GA	①③④⑩	All the included studies were of low quality (Jadad)	Compared to GA, acupuncture combined with traditional CM showed superior total efficiency, cure rate, and gastric emptying function in patients with DGP without increasing the incidence of adverse reactions. However, owing to the low quality of the included literature, more high-quality randomized controlled trials are needed in subsequent studies.

①, total efficiency; ②, improvement in dyspeptic symptoms; ③, adverse reactions; ④, gastric-emptying rate; ⑤, motilin secretion; ⑥, gastrin secretion; ⑦, single dyspeptic symptom; ⑧, recurrence rate; ⑨, small barium strip stomach residue; ⑩, cure rate; AC, acupuncture; AI, acupoint injection; CM, Chinese herbal medicine; DGP, diabetic gastroparesis; EA, electroacupuncture; GA, gastroprokinetic agents; MA, meta-analysis; MN, mango needles; ROB, risk of bias; SA, sham acupuncture; SR, systematic reviews; WA, warm acupuncture; a, random sequence generation (selection bias); b, allocation concealment (selection bias); c, blinding of participants and personnel (performance bias); d, blinding of outcome assessment (detection bias); e, incomplete outcome data (attrition bias); f, selective reporting (reporting bias); g, selective reporting (reporting bias).

### 3.3. Quality assessment

#### 3.3.1. Methodological quality assessment

**Figure 2:**
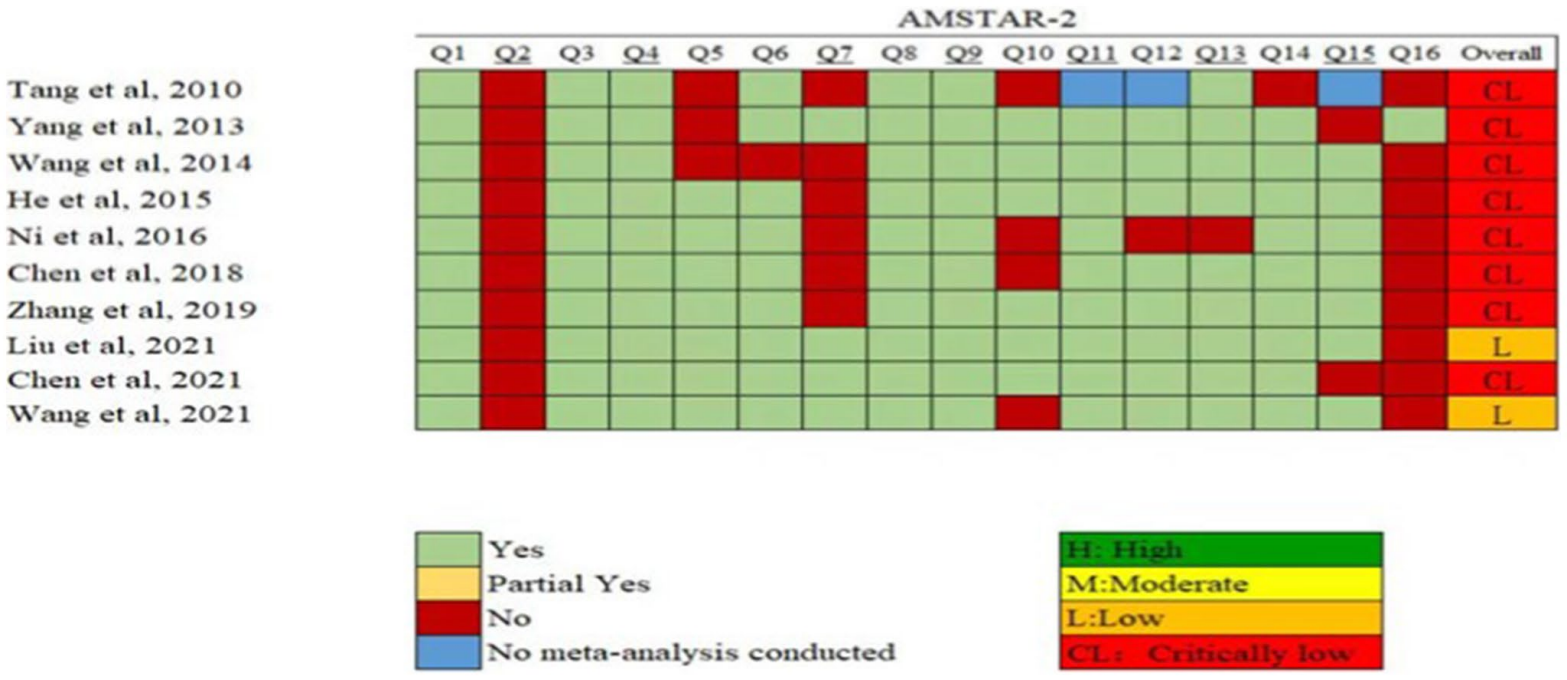
Quality of the methodology of the SRs/MAs included in this study determined by AMSTAR-2. AMSTAR-2: AMSTAR-2: Assessing the Methodological Quality of Systematic Reviews Two.

The results of the AMSTAR-2 assessment are shown in Figure 2. Two MAs were rated as low quality. The remaining seven MAs and one SR were rated as very low quality. The poor quality of the SRs/MAs was attributed to the following: all the SRs/MAs were not registered in advance (item 2); 90% of the SRs/MAs did not state whether conflicts of interest existed (item 16); 60% of the SRs/MAs did not state the criteria for excluding literature (item 7); 40% of the SRs/MAs did not indicate the source of funding (item 10); and 30% of the SRs/MAs did not clarify whether two individuals independently screened the literature (item 5).

#### 3.3.2. Quality of the results

The results of the GRADE assessment are shown in Table 2. In total, 20 results were evaluated, and 4 were rated as medium quality, 10 as low quality, and 6 as very low quality. Total efficiency was reported nine times, of which three evaluations were of medium quality and six were of low quality, and it was the most frequently reported result. Adverse reactions, gastric-emptying rate, motilin secretion, and gastrin secretion were reported twice each. Results pertaining to the gastric-emptying rate and motilin secretion were rated as very low quality. Gastrin secretion was reported twice and these results were rated low and very low quality once each. Adverse reactions were reported two times, and these results were rated as moderate and low quality once each. Improvement in gastric symptoms, small barium strip stomach residue, and cure rate were reported once each. Results pertaining to the improvement in gastric symptoms and cure rate were rated low quality, and the results pertaining to small barium strip stomach residue were rated very low quality. The major reason for the low quality of the results was the risk of bias (100%), followed by publication bias (50%), imprecision (40%), and inconsistency (30%).

**Table 2:** Quality of results determined using the Grading of Recommendations, Assessment, Development, and Evaluation tool.

Author	Outcomes	No. of trials	Limitations	Inconsistency	Indirectness	Imprecision	Other considerations	No. of treatments	No. of controls	Effect metric	Effect (95% CI)	Quality
Yang et al.	①	8	Serious	No	No	No	Serious	292	293	RR	1.20 (1.12, 1.29)	⊕⊕⊕○○ Low
	②	6	Serious	Serious	No	No	No	221	221	SMD	−0.99 (−1.29, −0.68)	⊕⊕⊕○○ Low
	④	6	Serious	Serious	No	Serious	No	195	195	SMD	−0.37 (−0.80, 0.05)	⊕⊕○○○ Very low
Wang et al.	①	9	Serious	No	No	No	No	372	306	OR	4.50 (2.76, 7.32)	⊕⊕⊕⊕○ Moderate
He et al.	①	10	Serious	No	No	No	Serious	361	348	OR	3.64 (2.35, 5.64)	⊕⊕⊕○○ Low
	③	3	Serious	No	No	Serious	No	154	150	OR	0.05 (0.02, 0.16)	⊕⊕⊕○○ Low
Zhang et al.	①	15	Serious	No	No	No	Serious	591	528	RR	1.33 (1.25, 1.42)	⊕⊕⊕○○ Low
Chen et al.	①	10	Serious	No	No	No	No	416	358	RD	0.18 (0.13, 0.23)	⊕⊕⊕⊕○ Moderate
Zhang et al.	①	8	Serious	No	No	No	No	325	313	OR	4.70 (2.94, 7.49)	⊕⊕⊕⊕○ Moderate
Liu et al.	①	16	Serious	No	No	No	Serious	643	511	RR	1.26 (1.20, 1.32)	⊕⊕⊕○○ Low
	⑤	4	Serious	Serious	No	Serious	No	184	185	MD	−28.00 (−45.92, −10.07)	⊕⊕○○○ Very low
	⑥	4	Serious	Serious	No	Serious	Serious	184	185	MD	−82.66 (−115.78, −49.55)	⊕○○○○ Very low
	⑦	3	Serious	No	No	Serious	No	140	140	MD	−3.11 (−3.43, −2.80)	⊕⊕○○○ Very low
Chen et al.	①	9	Serious	No	No	No	Serious	284	283	RR	1.24 (1.14, 1.34)	⊕⊕⊕○○ Low
	⑤	3	Serious	Serious	No	Serious	Serious	90	90	MD	−6.48 (−15.05, 2.08)	⊕○○○○ Very low
	⑥	4	Serious	No	No	Serious	No	135	134	MD	−11.97 (−15.86, −8.09)	⊕⊕⊕○○ Low
Wang et al.	①	20	Serious	No	No	No	Serious	738	713	RR	1.30 (1.23, 1.38)	⊕⊕⊕○○ Low
	③	6	Serious	No	No	No	No	225	224	RR	0.28 (0.10, 0.80)	⊕⊕⊕⊕○ Moderate
	④	7	Serious	Serious	No	Serious	Serious	150	150	MD	9.87 (8.90, 10.84)	⊕○○○○ Very low
	⑧	10	Serious	No	No	No	Serious	396	368	RR	1.55 (1.28, 1.88)	⊕⊕⊕○○ Low

①, total efficiency; ②, improvement in dyspeptic symptoms; ③, adverse reactions; ④, gastric-emptying rate; ⑤, motilin secretion; ⑥, gastrin secretion; ⑦, small barium strip stomach residue; ⑧, cure rate; CI, confidence interval; MD mean difference; OR, odds ratio; RD, risk difference; RR, risk ratio; SMD, standardized mean difference.

### 3.4. Evaluation of outcome indicators efficacy

#### 3.4.1. Total efficiency

As shown in Table 3, a total of eight interventions, i.e., acupuncture, electroacupuncture, AI, acupuncture combined with CM, electroacupuncture combined with CM, AI combined with CM, acupuncture combined with GAs, and AI combined with GAs, were re-analyzed in terms of total efficiency. The results indicated that acupuncture (OR = 3.77, 95% CI: 2.82–5.04, *p* < 0.001, *I^2^* = 0.0%, *n* = 25), electroacupuncture (OR = 3.01, 95% CI: 1.88–4.82, *p* < 0.001, I2 = 0.0%, *n* = 9), acupuncture point injections (OR = 4.49, 95% CI: 2.12–9.52, *p* < 0.001, *I^2^* = 0.0%, *n* = 3), acupuncture combined with CM (OR = 4.42, 95% CI: 3.29–5.94, *p* < 0.001, *I^2^* = 0.0%, *n* = 21), electroacupuncture combined with CM (OR = 3.93, 95% CI: 2.17–7.10, *p* < 0.001, *I^2^* = 0.0%, *n* = 5), acupuncture point injections combined with CM (OR = 4.10, 95% CI: 2.05–8.17, *p* < 0.001, *I^2^* = 6.3%, *n* = 4), acupuncture combined with GA (OR = 3.78, 95% CI: 2.23–6.42, *p* < 0.001, *I^2^* = 0.0%, *n* = 5), and acupuncture point injections combined with GA (OR = 5.71, 95% CI: 2.06–15.84, *p* = 0.001, *I^2^* = 0.0%, *n* = 2) were superior to controls in terms of total efficiency.

**Table 3:** Re-analyses of the efficacy of acupuncture as a treatment for diabetic gastroparesis based on the meta-analyses included in this study.

Trials	Treatment	Control	Sample (T/C)	Effect metric	Effect (95% CI)	*I* ^2^	Model	*p*-value
Total efficiency								
25	AC	GA	663/591	OR	3.77 (2.82, 5.04)	0%	Random	<0.001
5	AC + GA	GA	231/231	OR	3.78 (2.23, 6.42)	0%	Random	<0.001
21	AC + CM	GA	663/591	OR	4.42 (3.29, 5.94)	0%	Random	<0.001
9	EA	GA	309/308	OR	3.01 (1.88, 4.82)	0%	Random	<0.001
5	EA + CM	GA	150/150	OR	3.93 (2.17, 7.10)	0%	Random	<0.001
3	AI	GA	120/115	OR	4.49 (2.12, 9.52)	0%	Random	<0.001
2	AI + GA	GA	51/51	OR	5.71 (2.06, 15.84)	0%	Random	0.001
4	AI + CM	GA	175/168	OR	4.10 (2.05, 8.17)	6.3%	Random	<0.001
Adverse reactions								
3	AC	GA	154/150	OR	0.07 (0.02, 0.19)	0%	Random	<0.001
4	AC + CM	GA	153/150	OR	0.22 (0.07, 0.64)	0%	Random	0.006
Gastric-emptying rate								
3	EA	GA	90/90	SMD	−0.13 (−0.42, 0.16)	0%	Random	0.391
2	AC	GA	75/75	SMD	−0.22 (−0.58, 0.13)	5.5%	Random	0.21
4	EA + CM	GA	120/120	SMD	1.86 (0.42, 3.29)	95.3%	Random	0.011
3	AC + CM	GA	97/93	SMD	1.41 (0.91, 1.92)	59.3%	Random	<0.001
Motilin secretion								
4	EA	GA	134/135	SMD	−0.81 (−1.92, 0.3)	94.4%	Random	0.151
2	AI	GA	60/60	SMD	−0.47 (−0.84, −0.11)	0%	Random	0.011
Gastrin secretion								
4	EA	GA	134/135	SMD	−0.73 (−1.02, −0.43)	27.9%	Random	<0.001
2	AI	GA	60/60	SMD	−0.41 (−0.77, −0.05)	0%	Random	0.027

AC, acupuncture; AI, acupoint injection; CI, confidence interval; CM, Chinese herbal medicine; EA, electroacupuncture; GA, gastroprokinetic agents; OR, odds ratio; SMD, standardized mean difference; T/C, treatment/control.

#### 3.4.2. Secondary outcome indicators

The efficacy of four interventions with respect to gastric-emptying rates was re-analyzed. The results suggested that acupuncture combined with CM (SMD = 1.41, 95% CI: 0.91–1.92, *p* < 0.001, *I^2^* = 59.3%, *n* = 3) and electroacupuncture combined with CM (SMD = 1.86, 95% CI: 0.42–3.29, *p* = 0.011, *I^2^* = 95.3%, *n* = 4) were superior to the controls; however, significant heterogeneity was observed. The remaining two interventions, i.e., acupuncture (SMD = −0.22, 95% CI: −0.58–0.13, *p* = 0.21, *I^2^* = 5.5%, *n* = 2) and electroacupuncture (SMD = −0.13, 95% CI: −0.42–0.16, *p* = 0.391, *I^2^* = 0.0%, *n* = 3), were not markedly different from the controls in improving gastric-emptying rates. The efficacy of two interventions with respect to motilin and gastrin secretion was re-analyzed. The results showed that AI (SMD = −0.47, 95% CI: −0.84–−0.11, *p* = 0.011, *I^2^* = 0.0%, *n* = 3) significantly decreased motilin secretion compared to the controls. However, electroacupuncture (SMD = −0.81, 95% CI: −1.92–0.3, *p* = 0.151, *I^2^* = 94.4%, *n* = 4) was not significantly different from the controls in terms of motilin secretion reduction. Electroacupuncture (SMD = −0.73, 95% CI: −1.02–−0.43, *p* < 0.001, *I^2^* = 27.9%, *n* = 4) and AI (SMD = −0.41, 95% CI: −0.77–−0.05, *p* = 0.027, *I^2^* = 0.0%, *n* = 3) markedly decreased gastrin secretion compared to the controls.

#### 3.4.3. Adverse reactions

Eight of the ten articles included in this study reported adverse reactions, i.e., blood swelling, dizziness, and headache. The relevant RCTs were re-analyzed, and the results indicated that acupuncture (OR = 0.07, 95% CI: 0.02–0.19, *p* < 0.001, *I^2^* = 0.0%, *n* = 3) and acupuncture combined with CM (OR = 0.22, 95% CI: 0.07–0.64, *p* = 0.006, *I^2^* = 0.0%, *n* = 4) exhibited fewer side effects compared to the controls.

## 4. Discussion

### 4.1. Summary of the main findings

This study provides a comprehensive review of ten SRs/MAs of acupuncture as a treatment for DGP. The SRs/MAs were published between 2010 and 2021 and comprised a total of 126 RCTs and 9,270 participants. The methodology and results reported by the SRs/MAs were evaluated for quality using the AMSTAR-2 and GRADE tools. The primary outcome indicators were also re-analyzed by combining the included SRs/MAs. Results showed that, in the treatment of DGP, acupuncture is more effective compared to the controls. In addition, acupuncture was shown to have fewer side effects compared to the controls. The AMSTAR-2 tool was used to assess the methodological quality of the SRs/MAs. Two of the ten SRs/MAs were rated as low quality, and the remaining eight were rated as extremely low-quality. Using the GRADE tool, we evaluated 20 results obtained by combining the ten SR/MAs. The results showed that 20% of the results pertaining to the outcomes were rated as medium, 50% as low, and 30% as very low. Because the majority of the articles included in this study were determined to be of low quality, the findings on the effectiveness and safety of acupuncture as a treatment for DGP should be approached with caution. In the future, multicenter randomized controlled trials with a more rigorous experimental design and large sample sizes should be conducted to provide more reliable evidence for acupuncture as a treatment for DGP.

According to the results of the AMSTAR-2 quality assessment, the SRs/MAs included in this study had more than one weakness. In detail, most of the SRs/MAs were not registered; conflicts of interest were not declared; criteria for excluding literature were not provided; few of the articles did not indicate the source of funding; and whether literature was independently screened by two individuals was not clarified. These factors contributed to the poor-quality rating of the SRs/MAs. However, the main reason for the low rating was that the raw data measurements in the original studies exhibited a significant risk of bias owing to the flaws in the study design. In detail, most of the original studies did not adequately account for blinding, randomization, and allocation concealment. The reasons for the low rating of the outcome indicators were as follows: (1) the small sample size of the original studies had resulted in imprecision; (2) inconsistencies in the original studies because of differences in interventions and outcome indicators; and (3) publication bias in few of the articles as determined by Egger’s test.

In addition, to quantify the efficacy and safety of acupuncture as a treatment for DGP, we conducted a re-meta-analysis of five outcome indicators, namely total efficiency, adverse reactions, gastric-emptying rate, motilin secretion, and gastrin secretion. All the interventions, such as acupuncture, electroacupuncture, and AIs, were superior to the controls in terms of total efficiency. Compared to the controls, AI reduced motilin and gastrin secretion more effectively. Acupuncture and electroacupuncture combined with CM were superior to the controls in improving the gastric-emptying rate. However, electroacupuncture did not considerably differ from the controls in reducing gastrin secretion. Acupuncture and electroacupuncture did not differ notably from the controls in terms of improving gastric-emptying rates. Moreover, compared to the controls, acupuncture exhibited fewer side effects in the treatment of DGP.

Acupuncture includes general needling, electroacupuncture, and acupuncture point injection. Electroacupuncture is one of the key modalities of acupuncture for the treatment of DGP. Quantitative analysis has revealed that electroacupuncture exhibits increased total efficiency and is effective in reducing the secretion of gastrin. Studies have shown that the mechanisms underlying acupuncture as a treatment for DGP may be associated with the regulation of ICC, gastrointestinal hormone levels, intestinal flora, and enteric nerves. In detail, studies have shown that, first, acupuncture can treat DGP by upregulating the expression of SCF and c-kit in gastrointestinal smooth muscles and improving the number and morphology of ICC. In addition, electroacupuncture can inhibit ICC cell autophagy through relevant signaling pathways or modulate apoptosis-related factors, resulting in increased DGP gastric motility (29–32). Second, electroacupuncture can facilitate gastric emptying by inhibiting the secretion of motilin, gastrin, and cholecystokinin. Gastric emptying can be slowed by the combined action of motilin and gastrin. The secretion of cholecystokinin can also inhibit gastric emptying (33–35). Third, electroacupuncture can regulate intestinal flora and improve the function of the gastrointestinal tract in patients with DGP. Patients with DGP are prone to dysbiosis of the intestinal flora, with a decrease in intestinal probiotics and an increase in pathogenic enterobacteria, which predispose patients to gastrointestinal dysfunction (36, 37). Fourth, electroacupuncture can regulate the imbalance of excitatory and inhibitory neurotransmitters in the enteric nervous system and reduce the damage to enteric neurons. It also promotes the secretion of neurotrophic factors by glial cells, which helps boost the survival of enteric neurons, thereby facilitating gastric emptying. Patients with DGP are susceptible to the damage of enteric neurons and the enteric nervous system, and the loss of key neurotransmitters results in altered neurotransmission (38, 39).

### 4.2. Strengths and shortcomings

Although acupuncture as a treatment for DGP has been the subject of several SRs/MAs, this study is the first to provide an overview of the development of acupuncture as a treatment for DGP. In this study, to ensure the accuracy of the results, two researchers independently conducted the literature search, literature screening, data extraction, and quality assessment of the articles. To quantify the effectiveness of acupuncture as a therapy for DGP, we conducted a re-meta-analysis of relevant outcome indicators. Meanwhile, to ensure that our results were reliable, we screened all the included RCTs and excluded any duplicates.

This study has a few limitations. The majority of the articles included in this study were published in Chinese, and the study population was predominantly Chinese. No studies have been published on acupuncture as a treatment for patients with DGP from other countries or ethnicities. These factors are likely to contribute to the risk of publication bias in articles. Although a comprehensive search strategy was used in this study, there is no guarantee that all SRs/MAs of acupuncture as a treatment for DGP were retrieved. The low quality of the RCTs contributed to the low quality of the SRs/MAs, and this may have had an impact on the reliability of the results of this study. Therefore, the findings of this study should be approached with caution.

## 5. Conclusion

By re-analyzing previously published SRs/MA on acupuncture as a treatment for DGPs, this study showed that in the treatment of DGP, acupuncture exhibits more efficacy and fewer side effects compared to Western medicine. However, the quality of the methodology and results of the SRs/MAs examined in this study was determined to be low. Therefore, the findings of the SRs/MAs cannot be relied upon. In the future, to provide reliable evidence for the effectiveness and safety of acupuncture as a treatment for DGP, we should not only focus on the methodology employed in SRs/MAs but also establish more rigorous experimental protocols to improve the quality of RCTs in the original studies.

## Data availability statement

The original contributions presented in the study are included in the article/Supplementary material, further inquiries can be directed to the corresponding author.

## Ethics statement

Written informed consent was obtained from the individual’s primary caregiver for the publication of any potentially identifiable images or data included in this article.

## Author contributions

TL and BF planned and designed the study. MY and LH conducted the literature search. MY, LH, and TL screened the literature, extracted data from the included studies, assessed the quality, and data analysis. TL wrote the first draft. FS revised the first draft. All authors contributed to the article and approved the submitted version.

## Funding

This study was funded by the Shandong Provincial TCM Science and Technology Project (2020Q064).

## Conflict of interest

The authors declare that the research was conducted in the absence of any commercial or financial relationships that could be construed as a potential conflict of interest.

## Publisher’s note

All claims expressed in this article are solely those of the authors and do not necessarily represent those of their affiliated organizations, or those of the publisher, the editors and the reviewers. Any product that may be evaluated in this article, or claim that may be made by its manufacturer, is not guaranteed or endorsed by the publisher.
