# Supplementary material for: An overview of systematic reviews of acupuncture for diabetic gastroparesis
Source: Front Med (Lausanne). 2023 Jul 31;10:1196357. doi: 10.3389/fmed.2023.1196357 (PMC10424791; doi:10.3389/fmed.2023.1196357)

Supplementary Materials

Evaluation of Outcome Indicators Efficacy Forest plots

3.4.1 Total Efficiency Forest plots

3.4.2 Secondary Outcome Indicators Forest plots

3.4.3 Adverse Reactions Forest plots

3.4.1 Total Efficiency Forest plots

1. Acupuncture for DGP total efficiency forest plot


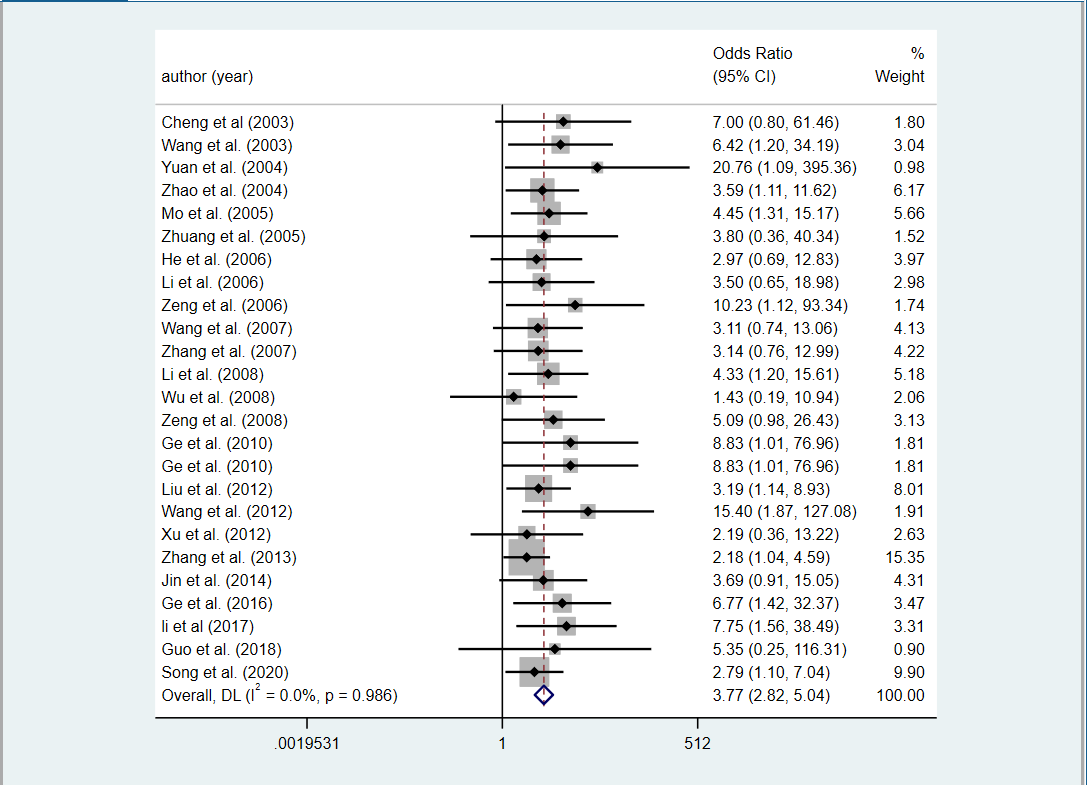


2. Acupuncture combined with gastroprokinetic agents for the treatment of DGP total efficiency forest plot


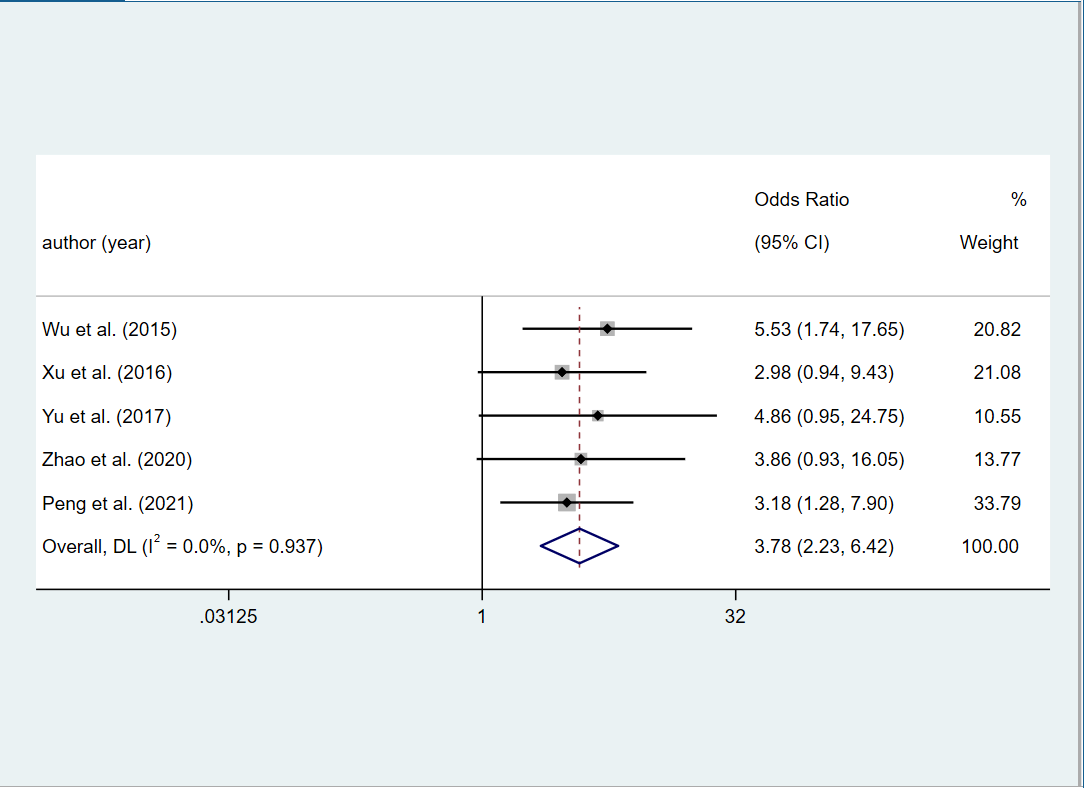


3. Acupuncture combined with Chinese herbal medicine for the treatment of DGP total efficiency forest plot


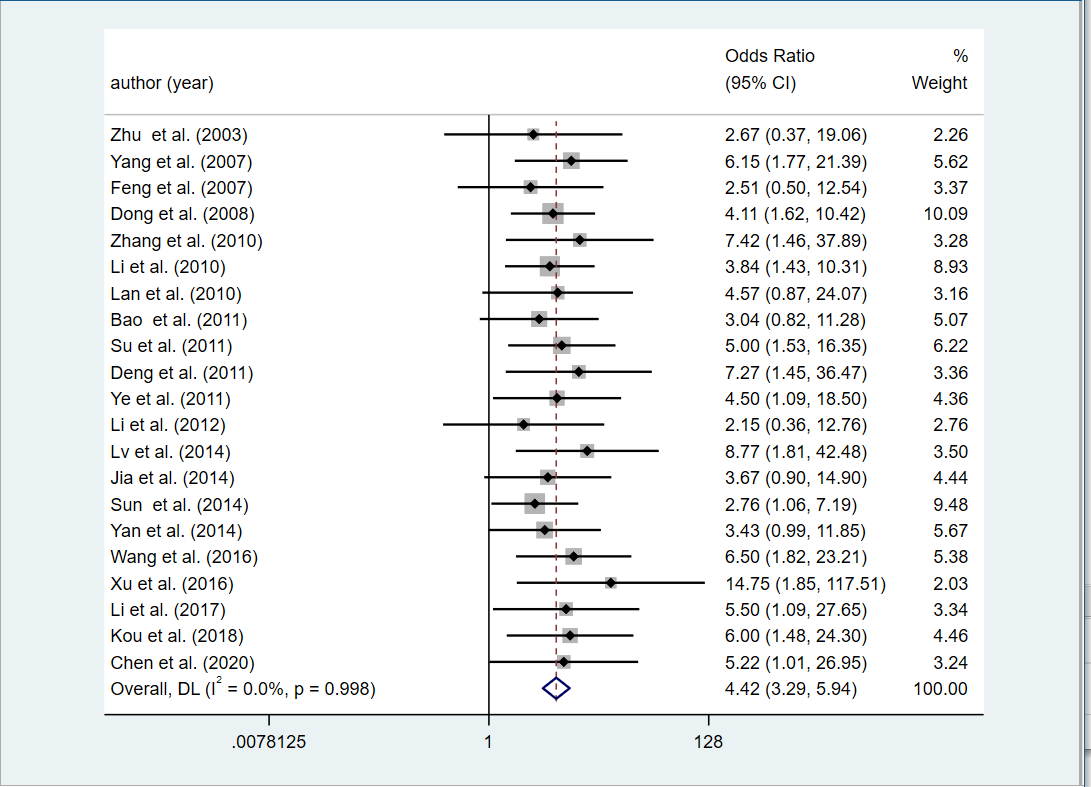


4. Electroacupuncture for the treatment of DGP total efficiency forest plot


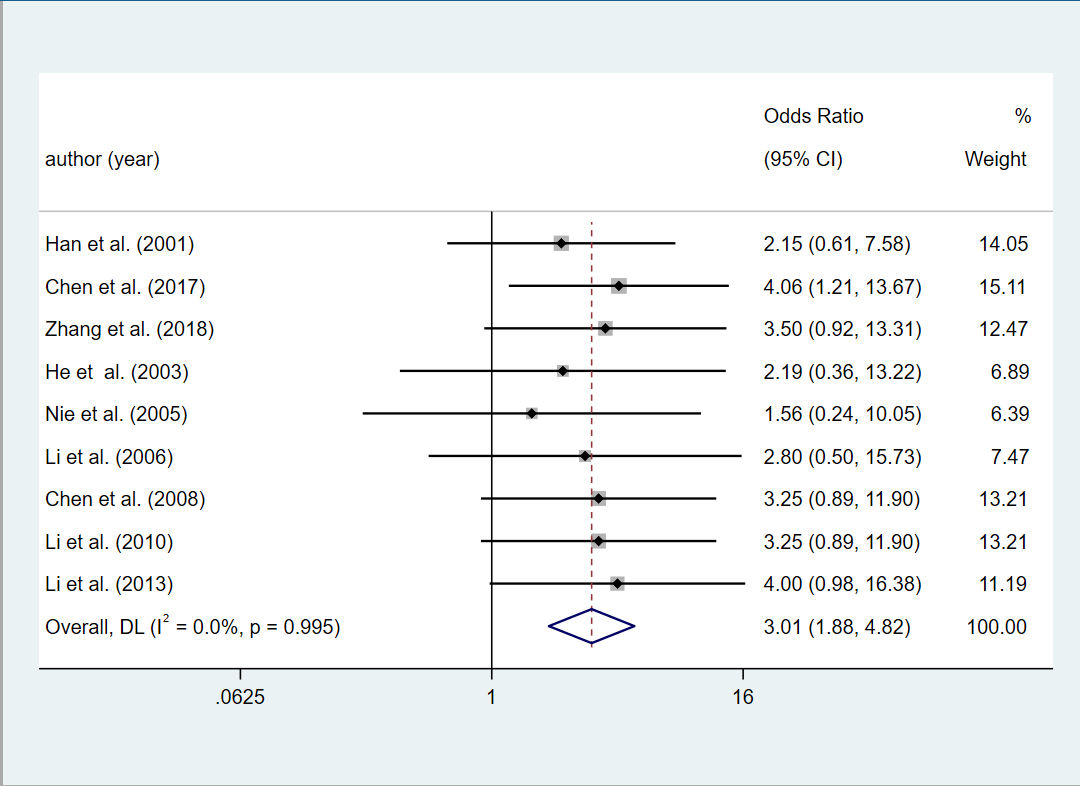


5. Total efficiency of electroacupuncture combined with Chinese herbal medicine for DGP forest plot


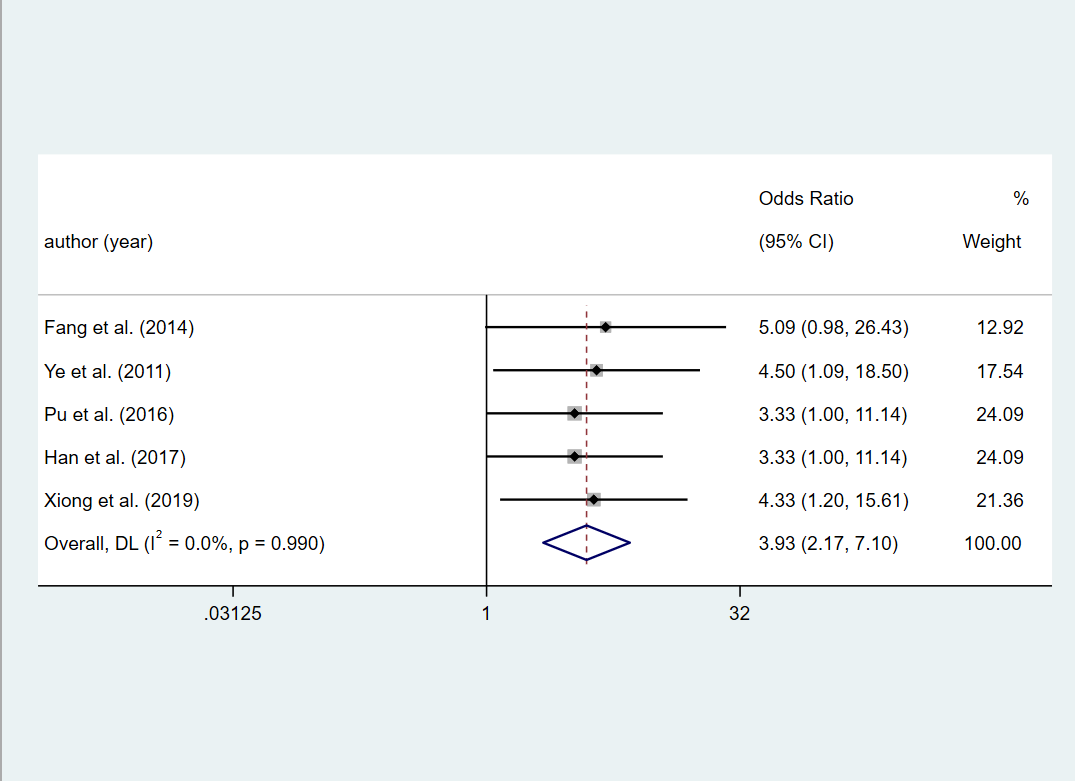


6. Acupoint injection for the treatment of DGP total efficiency forest plot


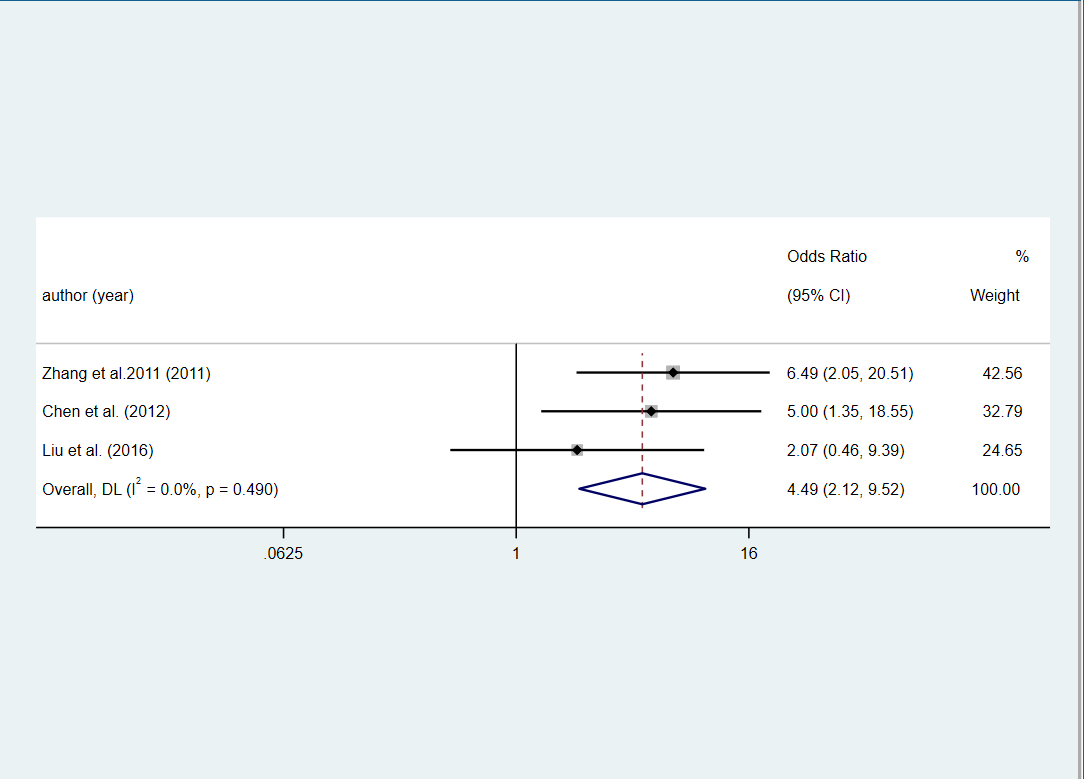


7. The total efficiency of acupoint injection combined with gastroprokinetic agents for DGP forest plot


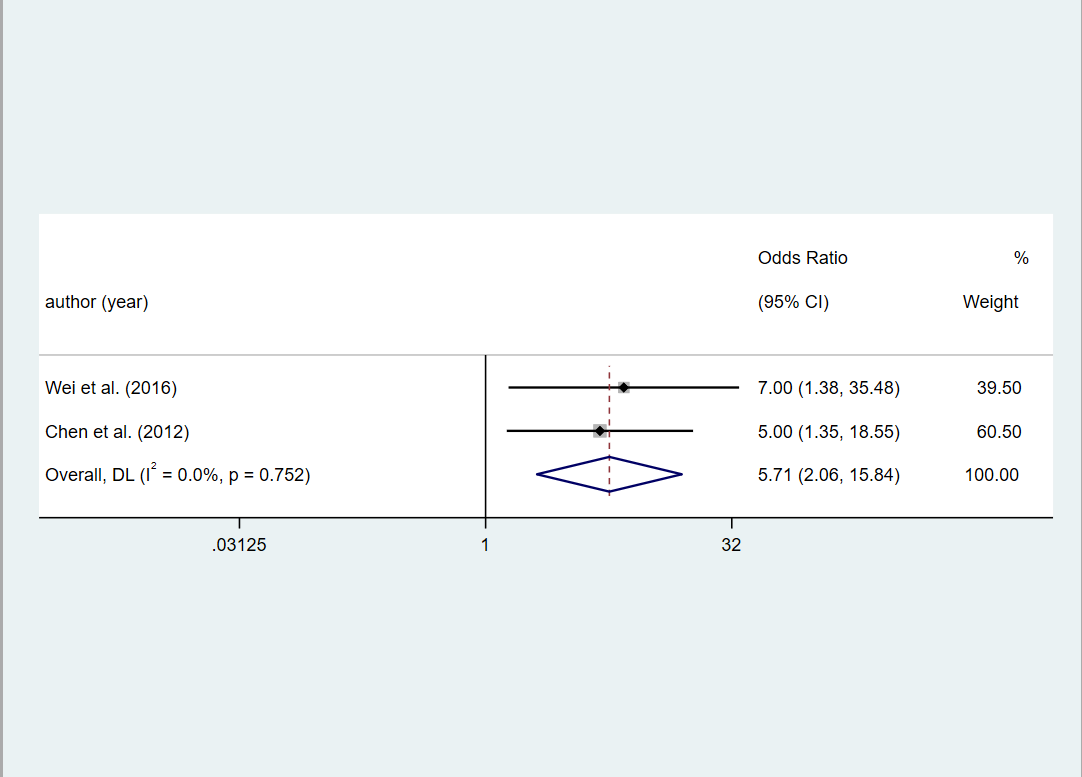


8. The total efficiency of acupoint injection combined with Chinese herbal medicine in the treatment of DGP forest plot


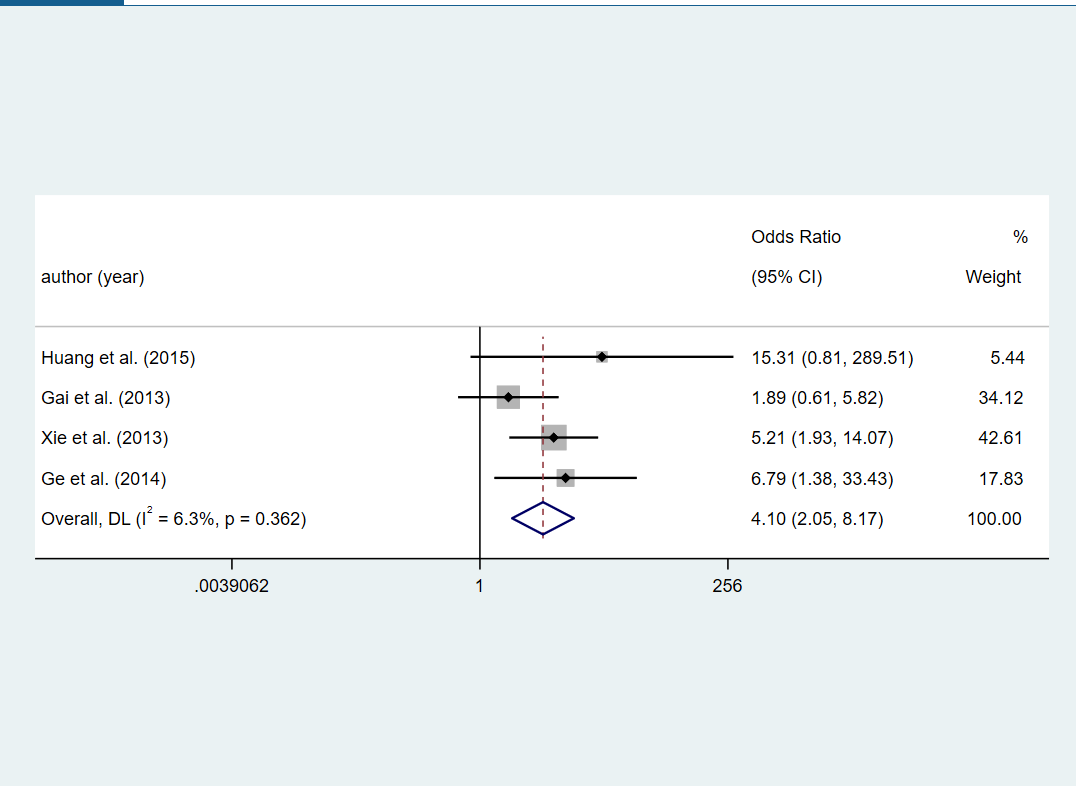


3.4.2 Secondary Outcome Indicators Forest plots

1. Electroacupuncture for DGP Gastric-emptying rate forest plot


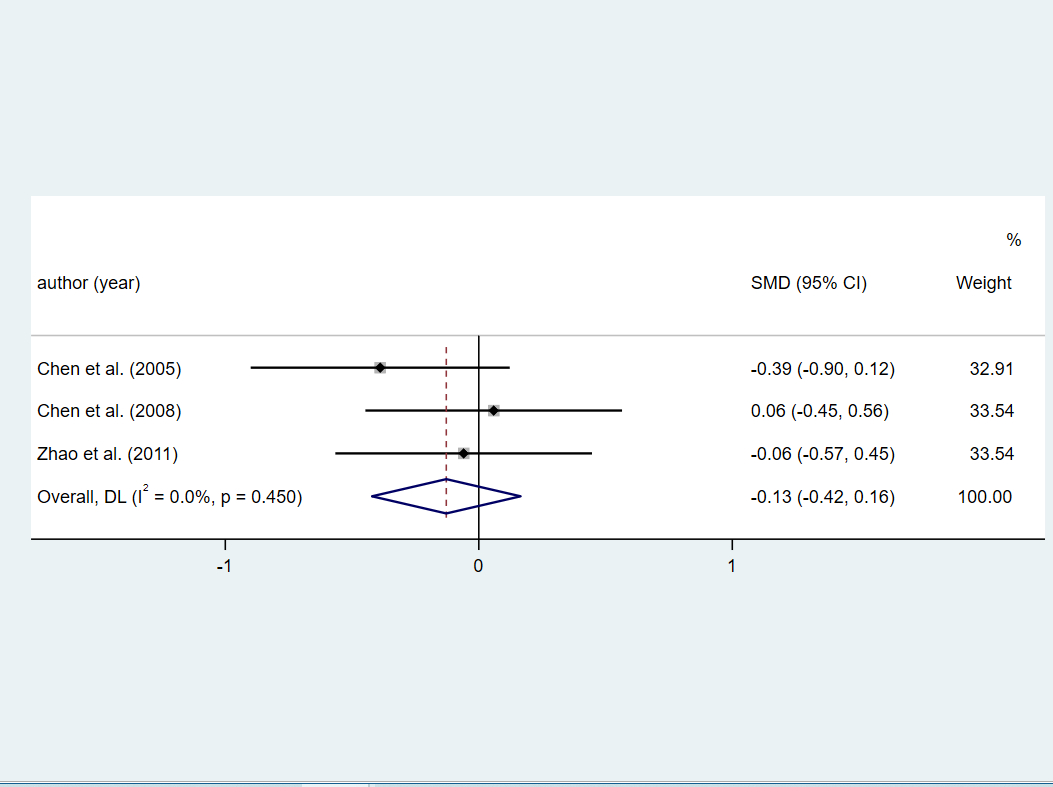


2. Acupuncture for DGP Gastric-emptying rate forest plot


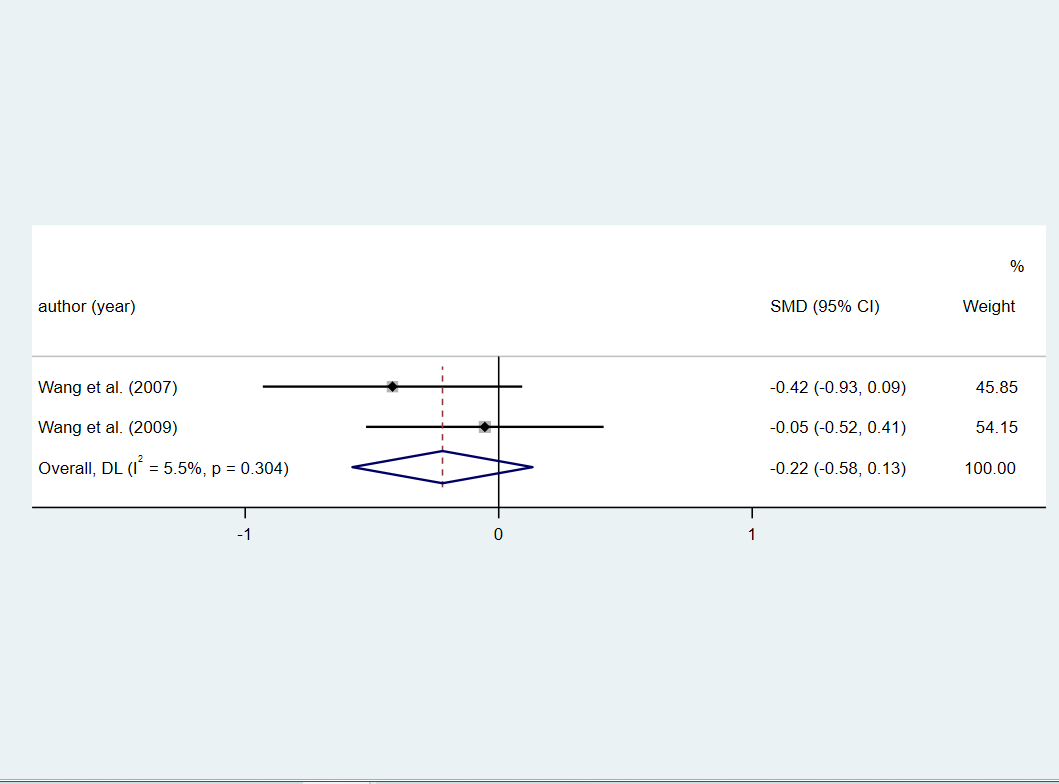


3 Electroacupuncture combined with Chinese herbal medicine for DGP gastric emptying rate forest plot


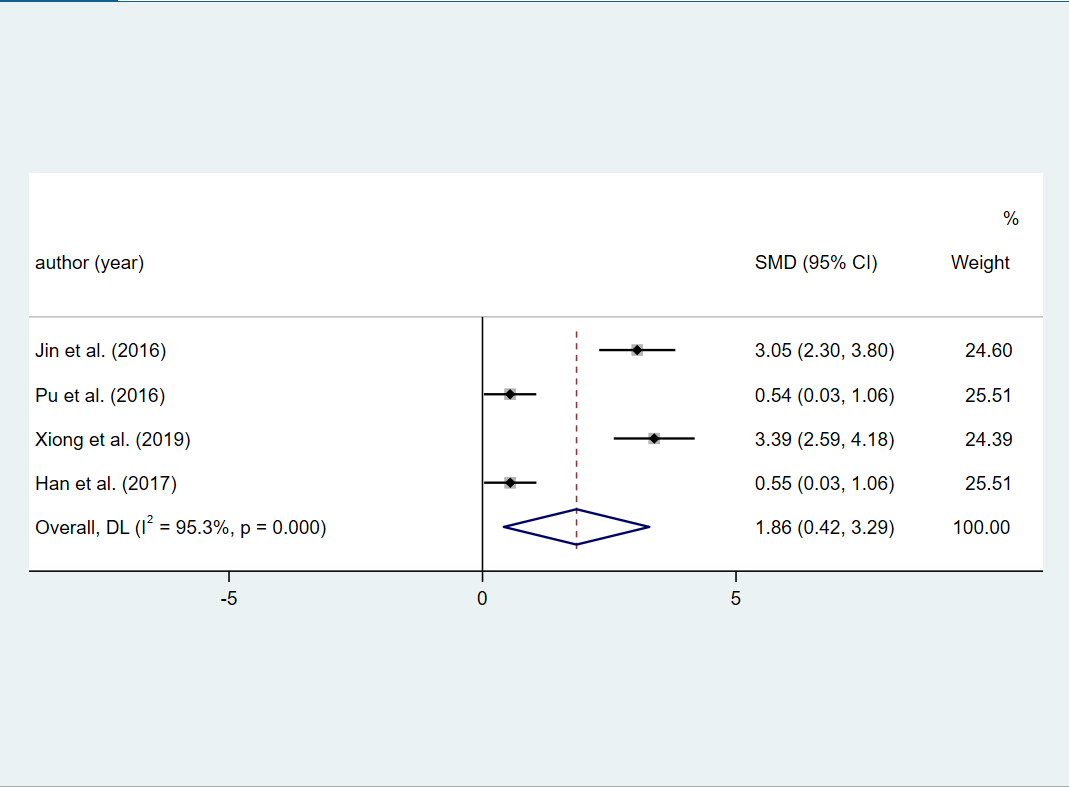


4. Acupuncture combined with Chinese herbal medicine for DGP gastric emptying rate forest plot


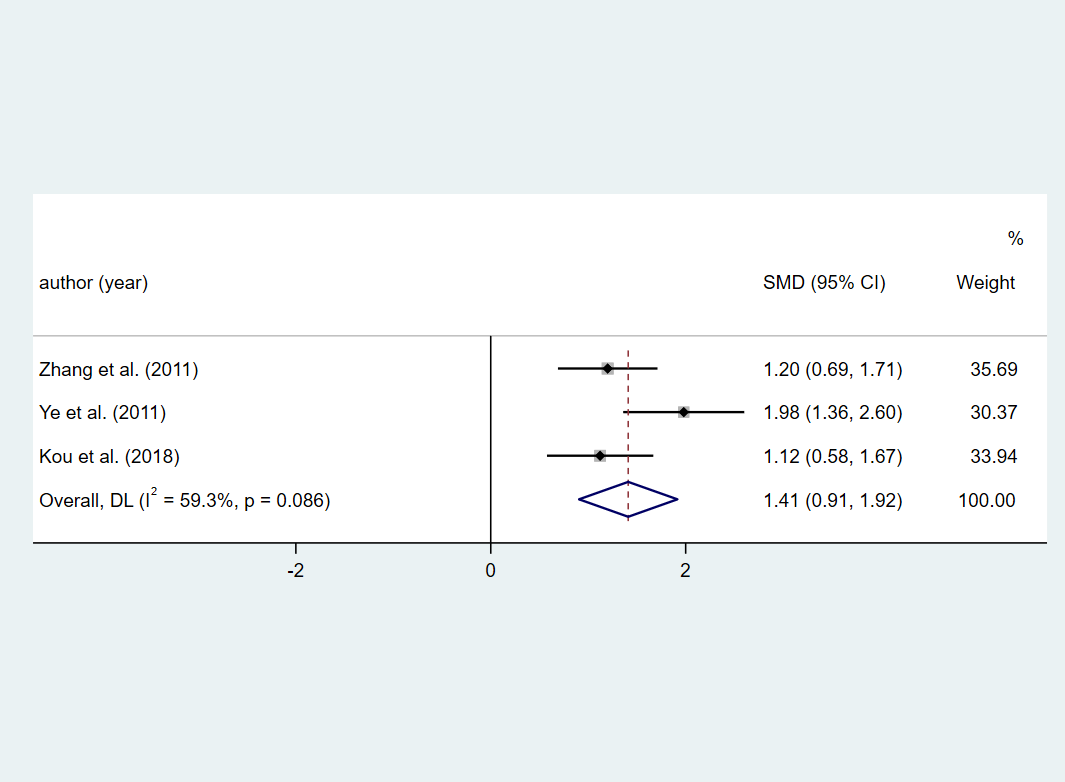


5. Electroacupuncture for DGP motilin secretion forest plot


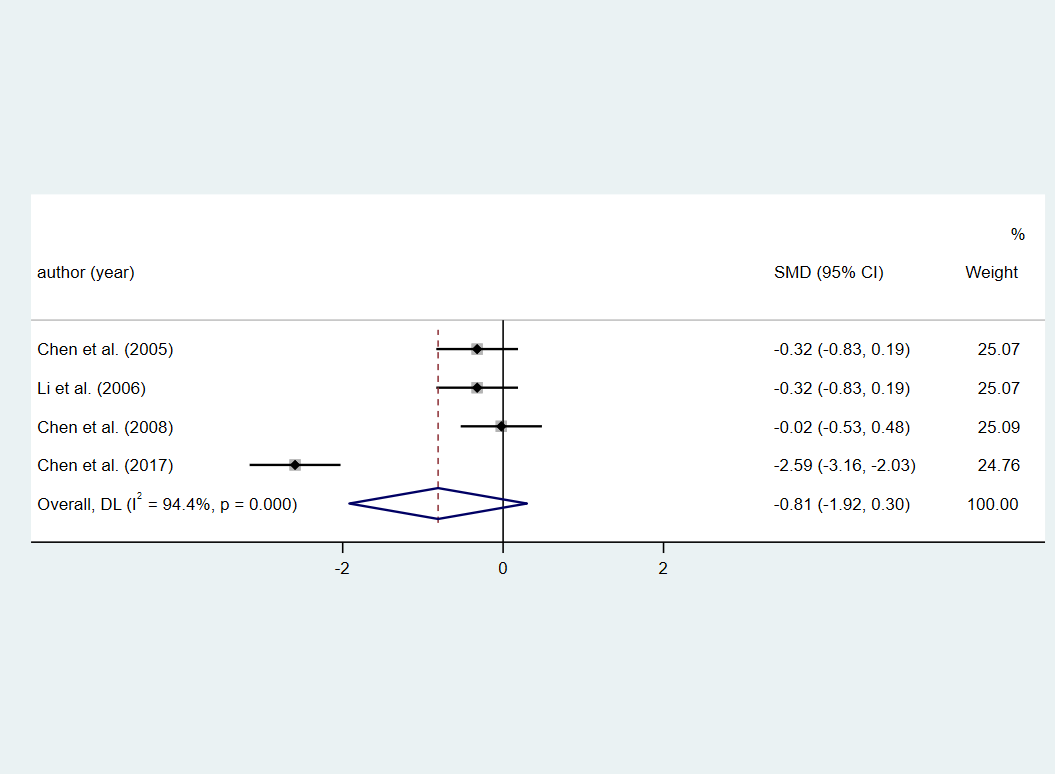


6. Acupoint injection for DGP motilin secretion forest plot


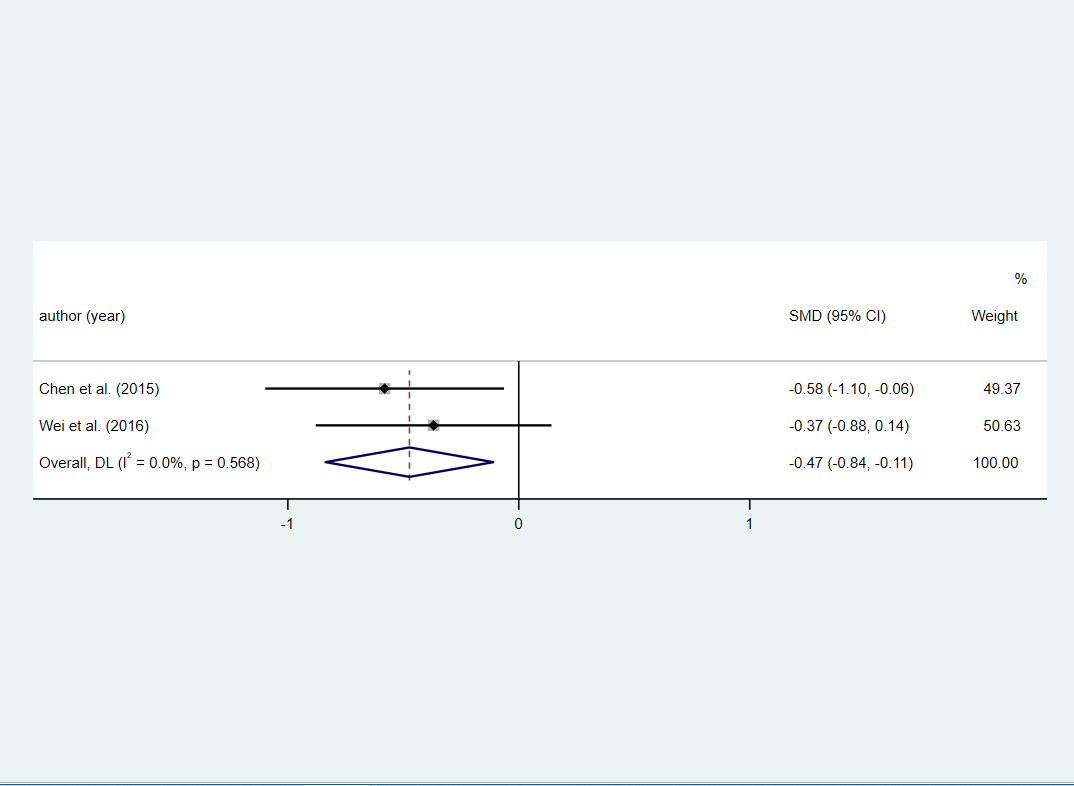


7. Electroacupuncture for DGP gastrin secretion forest plot


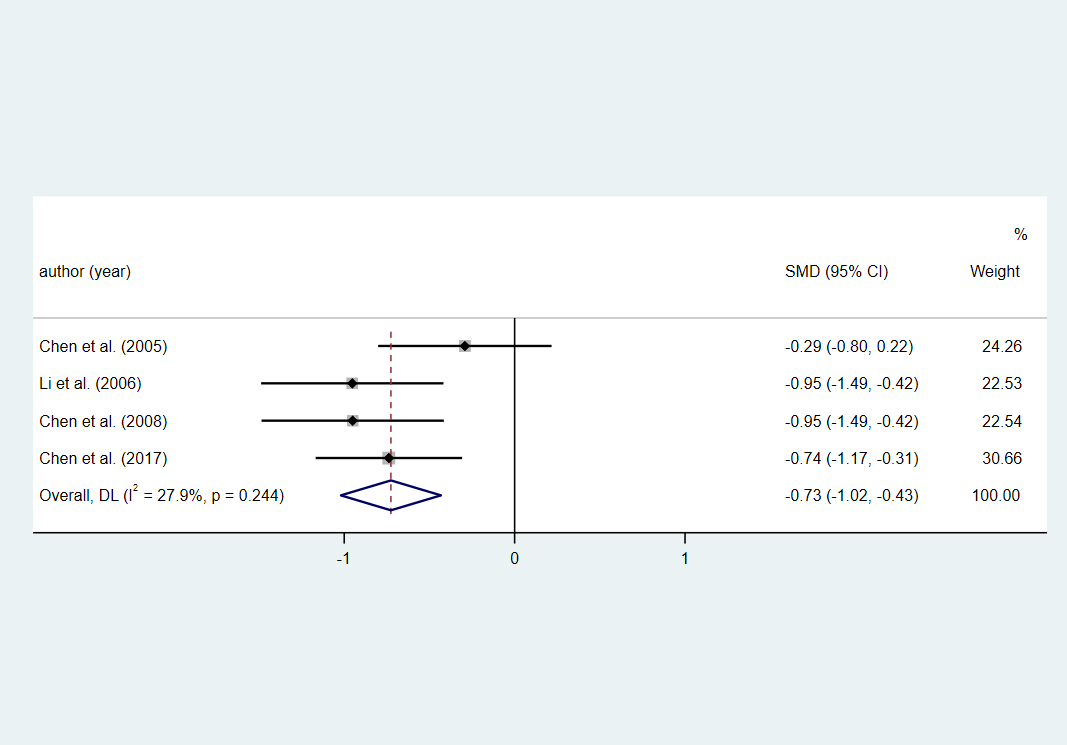


8. Acupoint injection for DGP gastrin secretion forest plot


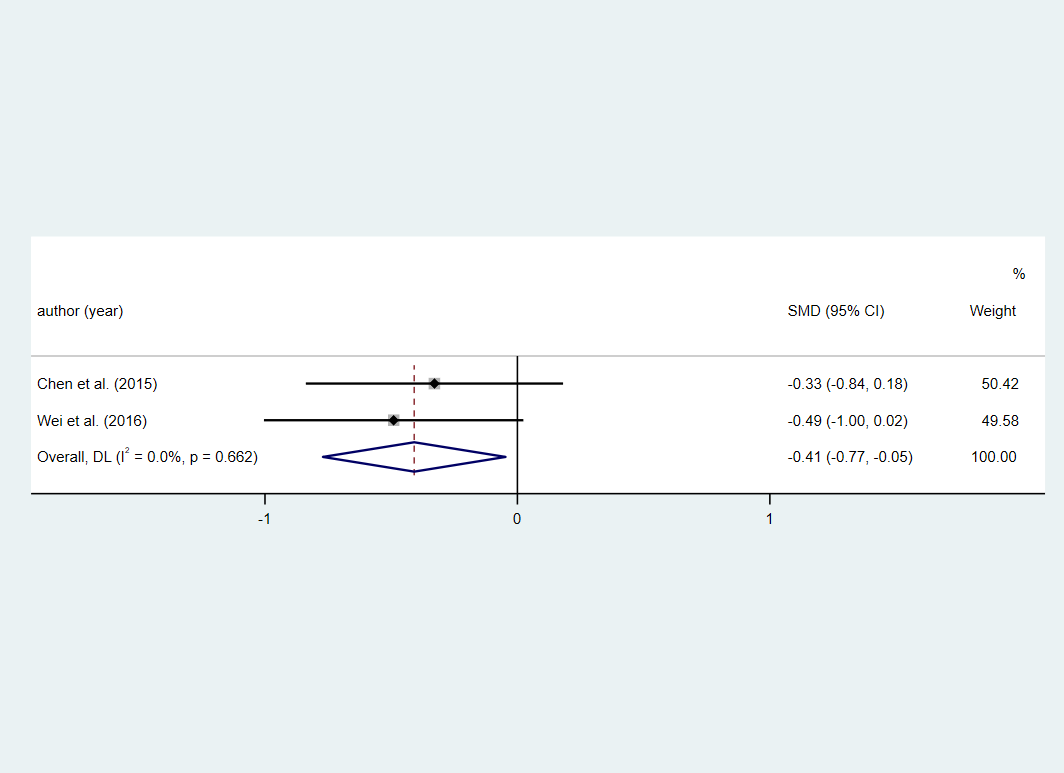


3.4.3 Adverse Reactions Forest plots

1.Acupuncture for DGP adverse effects P total efficiency forest plot


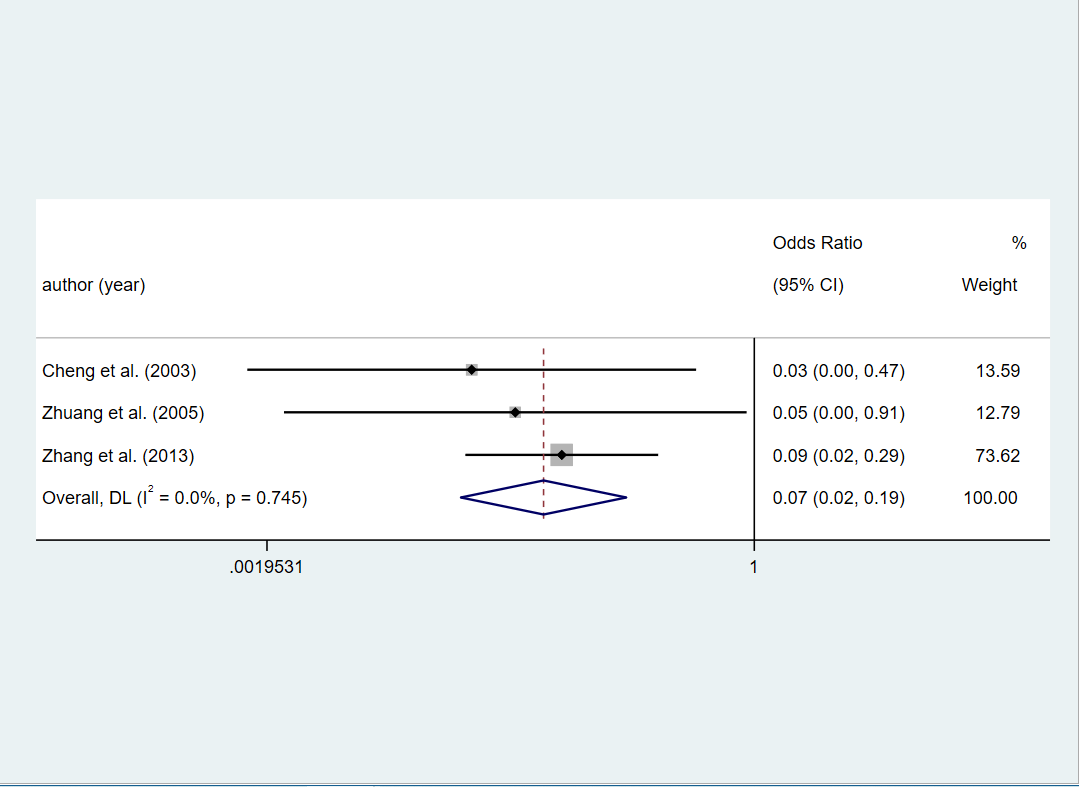


2. Acupuncture combined with Chinese herbal medicine for the treatment of DGP adverse reactions total effective rate forest chart


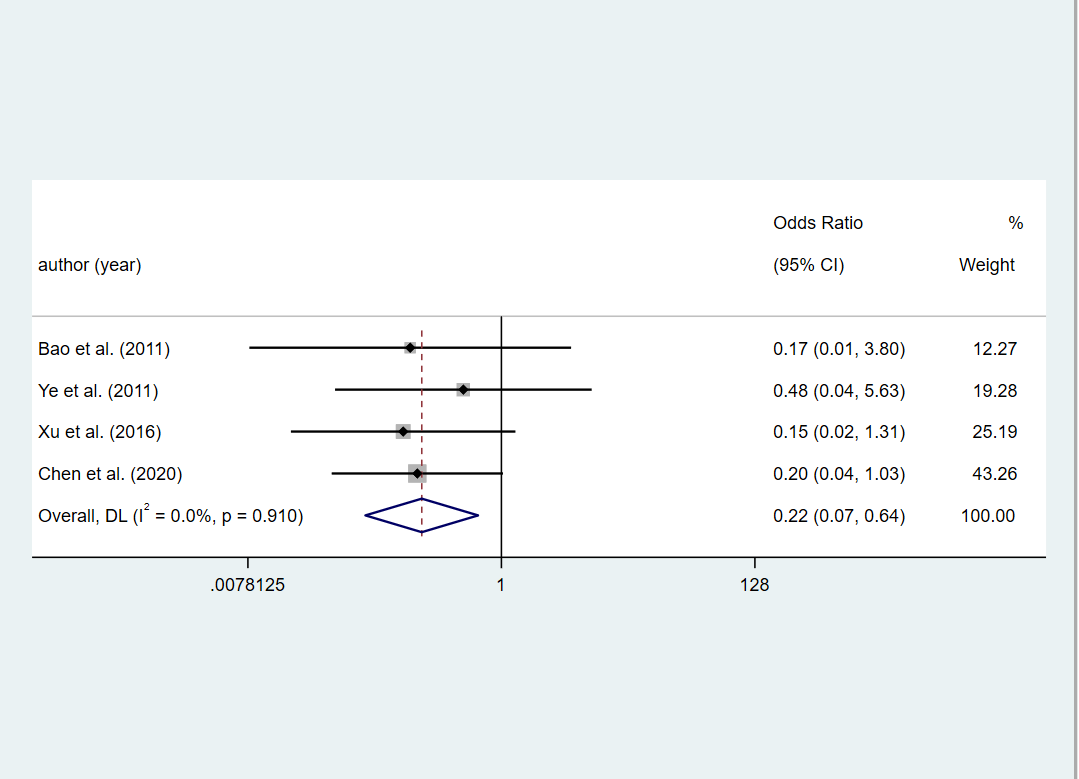

Supplement: Supplementary file 1 [file Data_Sheet_1.docx]
